# JNK and cardiometabolic dysfunction

**DOI:** 10.1042/BSR20190267

**Published:** 2019-07-19

**Authors:** Siobhan M. Craige, Kai Chen, Robert M. Blanton, John F. Keaney, Shashi Kant

**Affiliations:** 1 Division of Cardiovascular Medicine, Department of Medicine, University of Massachusetts Medical School, Worcester, MA 01605, U.S.A.; 2 Department of Human Nutrition, Foods, and Exercise, Virginia Tech, VA 24061, U.S.A.; 3 Department of Medicine, University of Connecticut Health Center, Farmington, CT 06030, U.S.A.; 4 Molecular Cardiology Research Institute, Tufts Medical Center, Boston, MA 02111, U.S.A.

**Keywords:** cardiovascular disease, metabolic regulation, Signaling

## Abstract

Cardiometabolic syndrome (CMS) describes the cluster of metabolic and cardiovascular diseases that are generally characterized by impaired glucose tolerance, intra-abdominal adiposity, dyslipidemia, and hypertension. CMS currently affects more than 25% of the world’s population and the rates of diseases are rapidly rising. These CMS conditions represent critical risk factors for cardiovascular diseases including atherosclerosis, heart failure, myocardial infarction, and peripheral artery disease (PAD). Therefore, it is imperative to elucidate the underlying signaling involved in disease onset and progression. The c-Jun N-terminal Kinases (JNKs) are a family of stress signaling kinases that have been recently indicated in CMS. The purpose of this review is to examine the *in vivo* implications of JNK as a potential therapeutic target for CMS. As the constellation of diseases associated with CMS are complex and involve multiple tissues and environmental triggers, carefully examining what is known about the JNK pathway will be important for specificity in treatment strategies.

## Introduction

The World Health Organization and the American Society of Endocrinology defines the term cardiometabolic syndrome (CMS) as a combination of different dysfunctions in our body which includes obesity, insulin resistance, dyslipidemia, and hypertension [1]. Approximately 25% of the world’s adults currently suffer from cardiometabolic dysfunction, and this number is rapidly expanding. With this in mind, it is paramount to uncover the underlying mechanism between these metabolic perturbations with the potential for targeted therapy in mind. The c-Jun N-terminal Kinase (JNK) pathway has been shown to be involved in cardiometabolic factors which include inflammation, insulin resistance, immune cell differentiation, and polarization. This review sets the stage for interrogation of the JNKs as possible therapeutic targets for cardiometabolic diseases, and to propose potential explanations for the discrepant phenotypes reported in experimental cardiovascular models.

**Figure 1:**
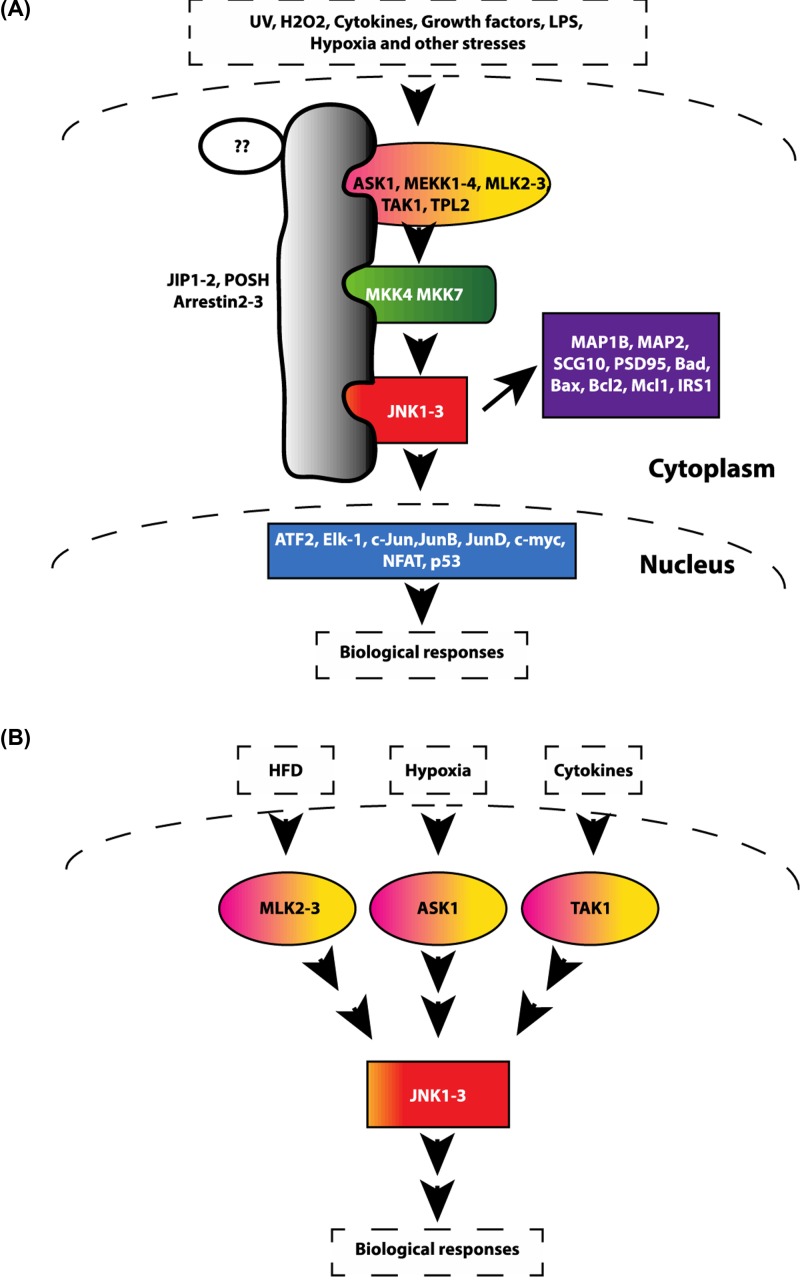
JNK pathway A schematic representation of the JNK pathway. (**A**) The JNK pathway can be activated by many stimuli, including UV, reactive oxygen species (ROS), growth factors, inflammatory cytokines, and a wide spectrum of cellular stresses. These stress signals then orchestrate the binding of multiple JNK-related proteins to scaffolding proteins (JIP1-2, POSH, Arrestin2-3). Upstream kinases phosphorylate and activate JNK which then phosphorylates different downstream targets, including protein kinases, cytosolic substrates, and transcription factors, leading to biological responses. (**B**) The JNK pathway can be activated by many stimuli, specificity comes from the selective activation of MAP3K activation by individual stimulus. Upstream kinases MLKs, ASKs, and TAK1 are activated mainly by high-fat diet (HFD), oxidative stresses, and cytokines, respectively. Abbreviations: ASK, apoptosis signal-regulating kinase; JIP, JNK-interacting protein; MLK, mixed lineage kinase; POSH, Plenty of SH3.

JNK was first discovered as a microtubule-associated protein kinase in the early 1990s [2,3]. Three separate genes encode the kinase, JNK1 (Mitogen Protein Kinase 8; *MAPK8*), JNK2 (*MAPK9*), and JNK3 (*MAPK10*), although alternative splicing can produce ten different protein sequences. There is much functional overlap in JNK1 and JNK2 signaling, while less is known about the third member, JNK3. While there remains much overlap in canonical JNK signaling (see Figure 1), we are still learning about the specificity in signaling among its members. In terms of expression, *JNK1* and *JNK2* are ubiquitously expressed, while the third member, *JNK3*, is principally expressed in the brain and nerves, and to a lesser extent, the heart, pancreatic islets, and testes. JNK3 studies have thus far largely focused on the function of JNK3 in the nervous system, and its role in neurodegenerative diseases like Alzheimer’s [4]. Currently, there is little knowledge about JNK3 in other tissue types.

JNK kinases can be activated by a number of external stimuli (e.g. cytokines, UV radiation, reactive oxygen species (ROS), heat shock, shear stress, and free fatty acids (FFA)) that contribute to its diverse role in a wide spectrum of biological processes affecting both nuclear and non-nuclear substrates [5] (Figure 1). The JNKs can directly bind and phosphorylate several transcription factors, including c-Jun, JunB, c-fos, ATF2, NFAT etc., that serve as the main mediators of JNK biological responses [6,7]. However, the specificity of these biological responses, which occur in the broad scope of JNK signaling, most likely stems from the tight upstream and downstream components of JNK signaling. Components included such as scaffolding proteins bring together a complex of specific JNK signal transducers. Below we discuss different scenarios that explain the coexistence of JNKs many functions and response specificity (Figure 1).

JNK-targeting stimuli trigger the activation of particular upstream kinases, MAP3Ks, including MLKs (Mixed lineage kinases), TAK1 (Transforming growth factor β-activated kinase 1), ASK (Apoptosis signal-regulating kinase), and MEKK (Mitogen-Activated Protein Kinase Kinase Kinase). Whereas MAP3K MLKs are mainly responsible for JNK activation by FFA [8–12], ASK plays a major role in ROS-induced JNK activation [13,14]. TAK1, another member of MAP3K family, plays an important role in cytokine-mediated JNK activation [15,16]. Activation of MAP3K kinases results in further activation of members of the MAP2K family (specifically MKK4 and MKK7) via phosphorylation (Figure 1). Both MKK4 and MKK7 activate JNK by phosphorylation on its conserved Thr-Pro-Tyr motif [3]. However, MKK4 prefers the Tyrosine residue for phosphorylation and MKK7 prefers the Threonine residue [17]. When phosphorylated, JNK modulates a large number of downstream substrates, including but not limited to the AP1 family of transcription factors (c-Jun, ATF-2, Jun-b, Jun-D, c-myc, NFAT1, NFAT4, Elk-1, MEF-2C, Smad4, Stat4, TCF, HSF1, DPC4, and p53), [3,7,18,19] (Figure 1).

JNK activity can also be counter-regulated by specific sets of phosphatases, enzymes that remove phosphate groups from proteins. Specifically, phosphatases MKP1, 2, 5, and 7 have been shown to control JNK activity by dephosphorylating JNK [20,21]. The current view holds that the specificity of JNK signaling stems from the ability of its JNK members to form complexes with different upstream kinases via various scaffold proteins such as the JNK-interacting proteins (JIP1, JIP2, JIP3, JIP4) [22], the Arrestins (2 and 3) [23–25], and Plenty of SH3 (POSH) [26] (Figure 1).

While there are functional redundancies between *Jnk1, 2*, and *3*, knockouts of a single *Jnk* member do not have the same phenotypes as double or triple -*Jnk* deficient animals. It is therefore appropriate to interpret these data from single knockout models cautiously, as other members or splice variants may have overlapping functions (Table 1) [27]. Alternatively, deletion of one JNK or JNK pathway regulator could potentially lead to up-regulation of different isoforms, making the resultant phenotype difficult to interpret. It is important to note that the alternative splicing of *JNK* isoforms have important implications for the interpretation of experimental results in different mouse models. As recent data show, *JNK1* and *JNK2* both have active and inactive splice variants, as well as phenotypes of knockout mice that can arise from the expression of differential splice variants in particular tissues [28]. For simplicity, unless specified, we will use the term JNK in reference to all three isoforms (JNK1, JNK2, and JNK3) in this review.

**Table 1:** Mouse models and their outcomes of JNK pathway deficiency

Disorder	Mouse model	Tissue	Outcome	References
Obesity/IR	*Jnk1^−/−^*	Whole body	JNK1-deficient mice were protected from obesity and insulin resistance	[29,30]
	*Mlk2^−/−^Mlk3^−/−^*	Whole body	The sympathoadrenal system contributes to the increased energy expenditure in MLK-deficient mice	[8, 9]
	*Jip1^−/−^*	Whole body	Jip deficiency decreased inhibitory phosphorylation of IRS-1 on Ser^307^ and therefore increased insulin sensitivity	[31]
	*Jnk1^−/−^*	Adipocyte	Mice with Jnk1-deficient adipocytes exhibit dramatically improved hepatic insulin sensitivity partially through less IL6 production by adipocytes	[30,32]
	*Jnk1^−/−^*	Liver	Promotes insulin resistance	[30,33]
	*Jnk1^−/−^ Jnk2^−/−^*	Liver	JNK-mediated inhibition of hepatic FGF21 promotes insulin resistance	[34]
	*Jnk1^−/−^*	Muscle	Reduced muscle LPL expression might contribute to the increased muscle insulin sensitivity in JNK1-deficient mice	[30,35]
	*Jnk1^−/−^*	Brain	Nervous system Jnk1 deficiency caused increased expression of thyrotropin releasing hormone (TRH) in the hypothalamus, thyroid stimulating hormone (TSH) by the pituitary gland, and increased circulating levels of thyroid hormones (T3 and T4) in the blood. Thus hypothalamic–pituitary–thyroid axis is a crucial target of JNK1 that controls obesity	[30,36,37]
	*Jnk1^−/−^ Jnk2^−/−^*	Anterior pituitary gland	JNK-dificient mice exhibited an increase in the pituitary expression of thyroid-stimulating hormone (TSH)	[38]
	*Jnk3^−/−^*	AgRP neuron	JNK3 deficiency causes hyperphagia selectively in high-fat diet (HFD)-fed mice	[39]
	*Jnk1^−/−^ Jnk2^−/−^*	Monocytes	JNK was necessary for pro-inflammatory macrophage (M1) polarization	[40]
	*Jnk1^*−/−*^*	Pancreatic b cells	JNK1 inhibits glucose-induced insulin production	[41]
Atherosclerosis	*Jnk1^*−/−*^*	Endothelium	JNK1 required for apoptosis and atherosclerosis	[42]
	*Jnk1^−/−^*	Bone marrow transplant	JNK1 deficiency promotes disease	[43]
	*Jnk2^−/−^*	Whole body	JNK2 deficiency reduced SR-A receptors endocytosis, therefore, less foam cell formation	[44]
Abdominal aortic aneurysm	*SP600125/Jnk2^−/−^*	Whole body	JNK2 deficiency reduced secretion of MMP-9 and MMP-2 from VSMCs, THP-1 macrophages	[45]
Myocardial infraction	*Jnk1^*−/−*^*		Pro-survival role when the period of ischemia is brief and injurious when the period of ischemia is extended	[46]
	*Ask1^*−/−*^*	Whole body	Protection	[47–49]
Peripheral artery disease (PAD)	*Jnk1^−/−^ Jnk2^*−/−*^*	Endothelium	JNK deficiency promotes PAD	[50]

## JNK links chronic inflammation to cardiometabolic diseases

Cardiometabolic diseases are characterized by a heightened inflammatory state, thought to promote disease and associated comorbidities [51–56]. JNK kinases have been thoroughly described for their role in cytokine signaling [8,57]. Data have demonstrated that cytokines can activate the JNK signaling pathway, but also that the JNK pathway is responsible for producing cytokines. The JNK kinases are responsible for sensing multiple stressors and reacting, ultimately resulting in proliferation, cell survival, or potentiating cell death if necessary via apoptosis. Under normal conditions these strategies serve to protect the organism by providing a platform for fast activation of downstream responses to an inflammatory stressor [8,27]. However, when homeostasis becomes dysregulated JNK activation is prolonged and results in aberrant apoptosis and chronic inflammation are mediated through prolonged cytokine production [30,51–56,58–62]. Therefore, JNK has an important role connecting the inflammatory response to the onset of multiple metabolic disorders [30,58,59].

Inflammation is a powerful biological response required to remove harmful agents from the body via both innate and adaptive immune responses. However, when the inflammatory response becomes chronically activated it can contribute to the onset of chronic diseases such as diabetes, obesity, and atherosclerosis [58,63]. The first evidence of the link between JNK and cardiometabolic inflammation is demonstrated by the JNK-dependent infiltration of immune cells (macrophages, T cells etc.) into adipose tissue and liver [63]. These macrophages were shown to be responsible for producing pro-inflammatory cytokines such as TNFα and IL6, ultimately resulting in impaired insulin signaling, insulin resistance (Table 1) [58,59,64,65], and cardiovascular diseases [56,66,67]. Under these conditions, JNK promoted cytokine production via AP1-driven gene transcription [68,69], thus triggering chronic inflammation. Furthermore, JNK upstream kinases MLKs and TAK1 promote cytokine production and inflammation (Table 1) [16,70]. Together these data indicate that targeting JNK may decrease chronic whole-body inflammation, thus, decreasing CMS onset and progression.

## JNK in inflammation and insulin resistance

JNK activation following a high-fat diet (HFD) results in inflammation, obesity, and insulin resistance (extensively reviewed in Solinas and Becattini [62]), all of which are risk factors for atherosclerosis and cardiovascular disease. JNK is critical for adipose tissue IL6 production, which is chronically elevated in mice after HFD and leads to impaired hepatic insulin signaling via STAT3–SOCS3 axis which leads to insulin receptor substrate 1 (IRS1) degradation [32]. In addition, JNKs seem to play a significant role in peripheral insulin resistance via cytokine production, inflammation and disruption of insulin signaling. JNK1 removal from hematopoietic compartments of mouse leads to the protection against HFD-induced insulin resistance by decreasing obesity-induced inflammation [71] which, by extension, may decrease risk for other chronic inflammatory diseases such as atherosclerosis.

In addition to increasing cytokine production by myeloid and other immune cells, JNK activation can control macrophage polarization and T-cell differentiation. [40,72]. Macrophages can be generally classified into two main categories according to their polarization: M1 (pro-inflammatory) and M2 (anti-inflammatory). The ratio of macrophage polarization determines the overall macrophage function and can affect disease conditions [54,55,73,74]. In adipose tissue, JNK is directly involved in controlling macrophage polarization. Consequently, this results in an increase in the M1 population leading to insulin resistance in mice on HFD [40]. This may also be particularly relevant in the development of atherosclerosis.

**Figure 2:**
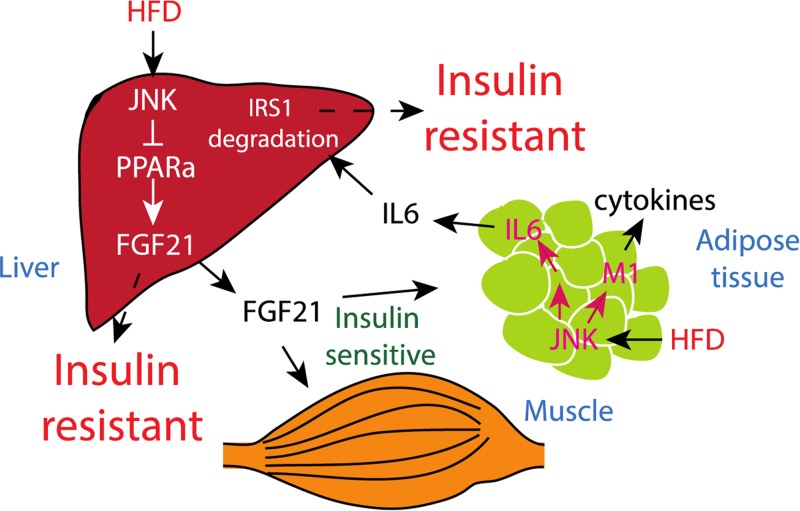
JNK controls insulin signaling JNK activation in the liver suppresses Fgf21 production, therefore reducing Fgf21 action on its target tissues, including liver, muscle, and adipose tissue, thus leading to insulin resistance. JNK activation also attenuates insulin signaling via increased Il6 production in adipose tissue, which then promotes IRS1 protein degradation in liver.

The liver can play an important role in cholesterol production and recycling as well as insulin signaling and gluconeogenesis. In the mouse liver, JNK plays a vital role in controlling the PPARa–FGF21 hormone axis which is required for fatty acid oxidation and ketogenesis [34]. In addition, PPAR has been exploited to treat hyperlipidemia and has been implicated in cardiovascular diseases in human [75,76]. Data from a recent study have shown that JNK in the liver tissue of mouse plays a critical role in insulin resistance via suppression of PPAR-controlled FGF21 pathway [34] (Figure 2).

Airways inflammation is a common phenomenon in chronic obstructive pulmonary disease (COPD). COPD is a co-morbidity of insulin resistance. A recent study examining insulin resistance of rat model showed that Wnt5a/JNK1 activation promoted macrophage activation and COPD [77].

Furthermore, upstream activators of the JNK pathway have recently been studied for their role in insulin resistance. Specifically, deficiency of the MAP3K MLKs or of the scaffold protein JIP, promotes insulin sensitivity in HFD-fed mice [9,31]. Collectively the data from these different studies support that the JNK pathway is required for insulin resistance mediated by multiple tissues (Figures 1 and 2).

## JNK in obesity

Obesity is strongly and independently associated with increased cardiovascular risks and mortality. In obesity, chronic inflammation is observed in the adipose tissue. Specifically, the visceral adipose tissue displays macrophage infiltration and acts as a secretory organ to introduce cytokines into circulation, potentially linking obesity to cardiovascular disease. Obesity is also associated with increased plasma FFA which are known to mediate many adverse metabolic effects leading to insulin resistance and atherosclerosis [78,79]. In cell culture, FFAs activate JNK [9] most likely through an activation of the upstream kinase, MLK, a member of the MAP3K family [10,12]. MLK is required for JNK activation in adipose tissue in mice on HFD (Table 1) [9]. Recently a scaffold protein JIP1 was shown to mediate a new pathway of FFA-induced JNK activation. Previously it has been shown that a non-receptor tyrosine kinase Src is also needed for FFA-induced JNK activation [11]. However, recent papers implicated that JIP1 is required for FFA-induced Src phosphorylation and its subsequent translocation to the membrane. Additionally, JIP tethers a Src–Vav–RAC1–MLK complex to FFA-dependent JNK activation [10]. This Src–Vav–RAC1–MLK pathway is required for obesity in mice as data have shown that mice with JIP, Vav, and MLK deficiency were protected from obesity after HFD feeding [9,31,80].

In addition, the visceral adiposity and vascular insulin resistance in the visceral adipose tissue arterioles of obese subjects were associated with increased JNK activation and impaired endothelial nitric oxide synthase (eNOS) activation. Pharmacological JNK inhibition (with SP600125), markedly improved insulin-mediated vasodilation and vascular endothelial function, further suggesting JNK as a potential target in obesity-related vascular diseases [81].

The above studies demonstrate that JNK plays an important role in obesity and insulin resistance [30]. But the bigger picture becomes more transparent from the conditional JNK1/2 -deficient mice in which selective deletion of JNK1/2 in neurons abolished the ability to gain weight after HFD feeding compared with the control mice [36]. It has been proposed that JNK plays an important role in the feedback loop of the T3 hormone to the hypothalamus, wherein, neural JNK1/2-deficient mice lack this feedback response; thus causing them to use more energy, leading to weight loss [30,36,38,82]. Although JNK1/2 deficiency in peripheral tissue induced insulin sensitivity, there was no significant difference in obesity in these mice [30,34,35]. On the other hand, the other family member, JNK3, is highly expressed in the brain. Unlike JNK1/2, JNK3 deficiency actually accelerates weight gain and obesity in mice after HFD feeding [39]. JNK3 deficiency was associated with enhanced excitatory signaling by AgRP neurons and hyperphagia [39]. These studies clearly show that JNK in the central nervous system is important in the regulation of HFD-induced obesity. Furthermore, these studies also reveal the potential different roles of JNK1/2 versus JNK3 in regulating CNS-dependent effects on weight gain (Table 1).

A recent study attempted to dissect the role of JNK signaling in this interorgan communication between adipose tissue and vessels. A transgenic mouse expressing a dominant-negative JNK (dnJNK) under the control of a P2 promotor, enabling adipocyte-specific expression of dnJNK, displayed decreased adipose tissue inflammation and circulating cytokines, as well as reduced early atherosclerosis staining [83]. Notably, visceral transplantation of dnJNK-expressing visceral tissue protected high-fat fed mice from inflammation and atherosclerosis. Further, administration of the adipocyte fatty acid binding protein (A-FABP), abolished the protective effect of dnJNK [83], indicating that the inflammatory effect of JNK in adipocyte is mediated by FABP. These studies indicate that tissue-specific JNK inactivation may help to delineate the role of JNK signaling in cardiometabolic diseases. Therefore, more studies are necessary to elucidate the *in vivo* relevance of these observations.

## JNK in atherosclerosis: complicated biology and divergent experimental results

Atherosclerosis is a highly complex, multi-step pathophysiological process that ultimately can lead to myocardial infarction or stroke. Atherosclerosis often occurs in conjunction with systemic inflammation and metabolic disease as both diabetes and obesity are known associated atherosclerotic risk factors. Atherosclerosis onset and progression adapts the following general sequence of events: (1) An initiating stimulus such as vascular injury, hypercholesteremia, or chronic inflammation results in endothelial dysfunction, activation, and/or apoptosis. This leads to increased vessel wall permeability to lipids and an increase in localized inflammation. Under these conditions, endothelial cells then become ‘activated’ meaning they express adhesion molecules that recruit monocytes and leukocytes to the area; (2) Monocytes then transmigrate across the blood vessel wall and differentiate into macrophages. As the vessel is lipid-laden, the macrophages ingest the low-density lipoproteins (LDLs) which gives them a foamy appearance, hence the term ‘foam cells’. These foam cells promote lesion progression by increasing localized inflammation and ROS production; (iii) T and B lymphocytes are recruited to the plaque and smooth muscle cells are recruited to the lumen and begin to proliferate and secrete collagen, elastin, and other extracellular matrix proteins.

**Figure 3:**
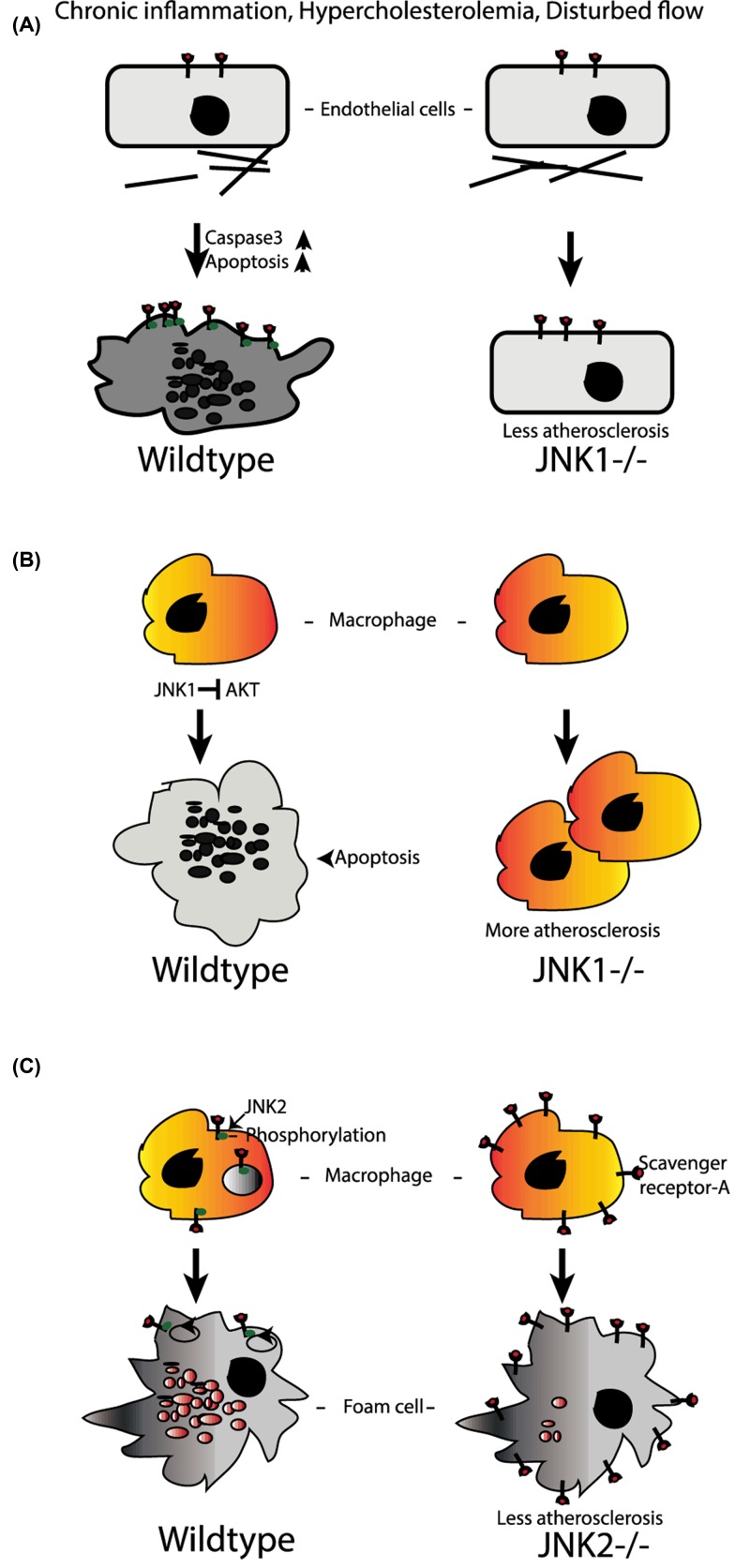
Role of JNK in atherosclerosis (**A**) JNK1 promotes apoptosis in endothelium after chronic inflammation and promotes atherosclerosis. (**B**) Similar to endothelium, JNK1 in bone marrow-derived immune cells (including monocytes) also promotes apoptosis after chronic inflammation which leads to less atherosclerosis in mice. (**C**) On the other hand, JNK2 knockout mice were protected from atherosclerosis through reduced number of foam cell formation as internalization of scavenger receptor A and lipid accumulation without phosphorylation of receptor are severely decreased.

While multiple cell types contribute to this complex process, many current lines of evidence implicate JNK 1 and JNK2 activation in each of these steps (Figure 3). As mentioned previously, JNK1/2 plays an important general role in inflammation and cytokine production. JNK1/2 is expressed in all of the cell types relevant to the onset and progression of atherosclerosis: endothelial cells, smooth muscle cells, macrophages, and T cells. Elevated JNK activity has been implicated in the formation and progression of atherosclerotic lesions [84] making JNK signaling critical to understand in this disease setting.

Initial *in vivo* studies using a mouse model of atherosclerosis, the Apo lipoprotein E knockout mouse (ApoE^−/−^) [44] in combination with either global Jnk1 deletion of global Jnk2 deletion, demonstrated that JNK2 independently promoted atherosclerotic lesion formation. The present study validates that JNK2 phosphorylation is essential in the development of foam cells as JNK2 phosphorylates the scavenger receptor-A (SR-A), which is required for lipid uptake by macrophages, resulting in the formation of foam cells (Figure 3). Inhibition of lipid uptake in the absence of JNK2 results in less foam cell formation and mitigation of atherosclerosis progression in the Jnk2^−/−^ ApoE^−/−^ mice.

Another recent study has shown that JNK deficiency in hematopoietic compartment increases the atherosclerotic lesion in Ldlr^−/−^ mice. In the present study macrophages were protected from apoptosis which accelerates the atherosclerosis in JNK-deficient mice [43].

As mentioned above, disturbance in the monolayer of cells that line the vasculature endothelium represents an early event preceding overt atherosclerosis. The endothelium can be injured by multiple atherosclerotic risk factors, such as hypertension, hypercholesterolemia, and environmental factors including cigarette smoke [85,86]. Chronic inflammation can contribute to endothelial dysfunction which can trigger multiple cardiometabolic diseases [87]. Endothelial dysfunction as demonstrated by inability of the endothelium to relax in response to stimuli and compromised nitric oxide bioavailability [88,89] is linked to insulin resistance, inflammatory activation, and is a significant predictor of cardiovascular events [90]. Recent studies in humans have demonstrated that JNK activation is associated with endothelial dysfunction as assessed by flow-mediated dilation (an established measure of endothelial function) [91]. Both cytokines and ROS activate JNK signaling and cause endothelial dysfunction [92], apoptosis, and endothelial ‘activation’ which promotes monocyte adhesion in areas susceptible to atherosclerotic plaque formation. With endothelial health in mind, the importance of JNK1 deletion was investigated in the LDL receptor knockout (LDLR^−/−^) model of atherosclerosis [42]. This study observed that loss of JNK1 protected in early atherosclerosis as it prevented induction of endothelial apoptosis (a characteristic of areas having disturbed flow in hypercholesteremic conditions) [42,93] (Figure 3).

As JNK plays multiple roles and is expressed widely in the tissues responsible for atherosclerosis, the specificity of the activation and resultant outcomes of JNK signaling likely involve the stimulus and upstream activators/repressors. As detailed in Figure 1, multiple upstream kinases and phosphatases function to regulate JNK signaling. One of the negative regulator of JNK, MAP kinase phosphatase-1 (MKP-1), has been investigated in several different models of atherosclerosis with mixed results. MKP-1 has been identified to prevent endothelial activation in sites resistant to atherosclerosis [94] suggesting that deletion would augment disease. However, two different studies demonstrated that MKP 1 deletion in ApoE mice protected mice from atherosclerotic lesions [95,96]. One study demonstrated stromal cell-derived factor-1a (SDF-1a), a factor negatively correlated with atherosclerotic lesion size, was increased in the serum with MKP-1 deletion [95] while the other study highlighted the decrease in monocyte chemoattractant protein-1 (MCP-1) and decreased capability of monocyte migration *in vitro* [96]. By contrast, a more recent study in LDLR^−/−^ mice identified that MKP-1 deficiency increased lesion formation through it effects on the macrophage phenotype. MKP-1 was required for macrophage polarization from M1 (inflammatory) to M2 (anti-inflammatory). In atherosclerosis, the macrophage M2 phenotype is inversely correlated with lesion size [97]. Therefore, this study indicates that JNK may also promote atherosclerosis development by influencing macrophage polarization (M1/M2 ratio) as discussed above. However, the specific links between JNK-mediated macrophage polarization and atherosclerosis remain unstudied. Some discrepancies in these studies may be due to different background of the mice (ApoE vs Ldlr) studied in these experiments as well as the possibility that JNK may play a different role in different cells and tissue types (macrophages, T-cells, endothelial cells etc.). Temporal and tissue-specific conditional knockout models will likely help clarify the specific role(s) of JNK in the complex process of atherosclerosis.

## JNK and abdominal aortic aneurysms

One disease that often coexists with atherosclerosis is the development of abdominal aortic aneurysm (AAA). Typically occurring in smokers and men over the age of 65, these aneurysms are the most common arterial aneurysms and can be fatal if not diagnosed. The localized dilation of the abdominal aorta involves vascular smooth muscle hypertrophy and chronic inflammation. JNK has been implicated as a critical molecular target in AAAs as human aneurysm tissue has shown a high level of phosphorylated (activated) JNK [45]. Pharmacological JNK inhibition in two different mouse models of AAA not only decreased the onset of aneurysms, but also caused regression of pre-existing aneurysms [45]. Taken together, these data support JNK as a critical player in the role of inflammation-related vascular diseases like atherosclerosis and AAA, indicating Jnk may be a robust therapeutic target for disease prevention.

## Cardiac hypertrophy and failure

**Figure 4:**
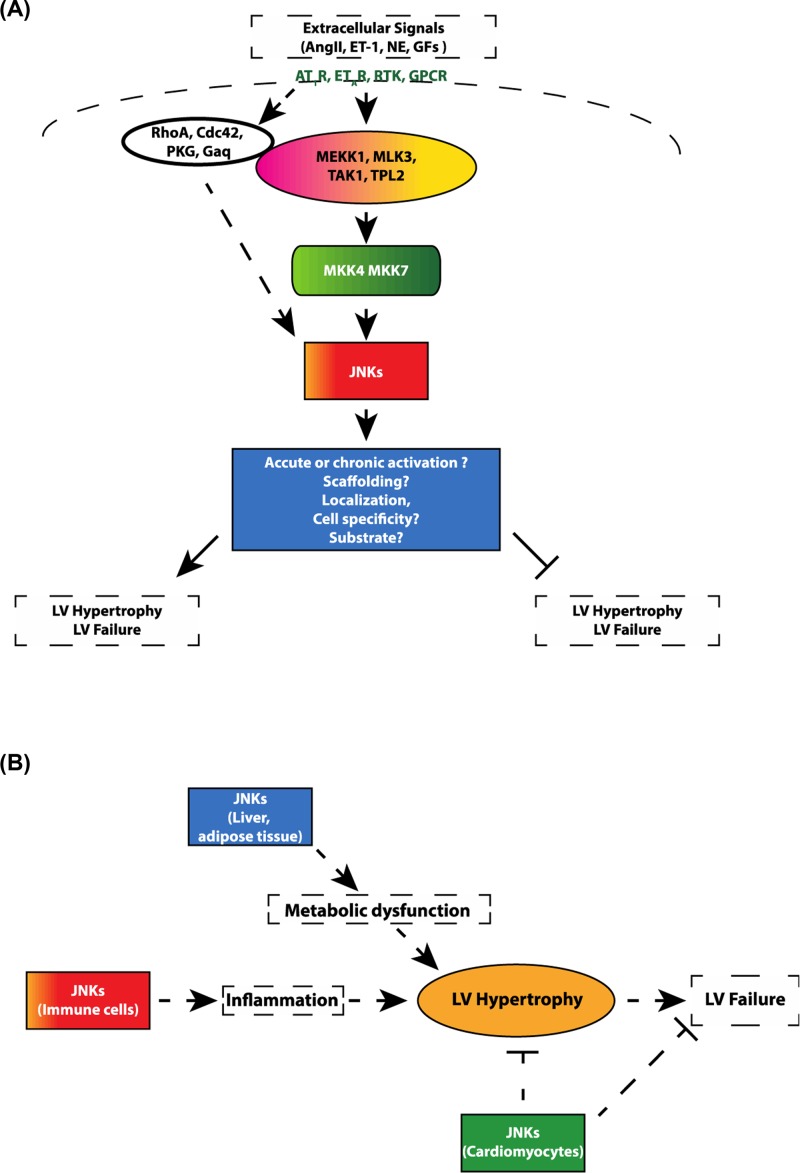
Proposed mechanisms through which JNK signaling promotes divergent hypertrophic phenotypes (**A**) Extracellular signals activate separate upstream MAP3Ks and JNK activators, leading to activation of MAP2Ks MKK4 and MKK7. Depending on localization of MAP2K, mediated by specific scaffolding proteins, JNK phosphorylates anti- or pro-hypertrophic signaling pathways. (**B**) JNK can play direct or indirect role in LV hypertrophy and heart failure.

Cardiac hypertrophy is the enlargement and/or thickening (hypertrophy) of the heart muscle that can occur in response to multiple stimuli such as mechanical stress, scarring, inflammation, and neurohumoral stimulation. Transient increases in heart size occur as an adaptive process. However, pathological stresses induce maladaptive hypertrophy associated with fibrosis, inflammation, oxidative stress, and ER stress, ultimately resulting in heart failure, arrhythmias, and potentially sudden death making it a significant health concern [98]. The JNK kinases have been implicated in multiple parts of these pathways (Figure 4) involved in cardiomyocyte growth and fibrosis. JNK activation increases in the human failing heart [99], and MLK an upstream JNK activator expression becomes elevated in end-stage heart failure patients [100]. JNKs are thought to be involved in re-programing gene expression that, in part, results in hypertrophic gene expression through regulation of transcription factors such as NFATs, Stats, Creb, c-jun, c-fos, and Gata4. In addition, in multiple contexts, the JNK family both initiates and propagates chronic inflammation which promotes and exacerbates pathological cardiac hypertrophy [101]. Further, multiple studies in cultured neonatal cardiac myocytes (CMs) identified that JNK promoted myocyte growth and the fetal gene expression pattern which marks pathologic hypertrophy [102–104]. Taken together, these correlative studies in human patients and studies in cultured CMs have raised interest in better understanding the biology of JNK signaling in the process of LV hypertrophy and failure. As JNK signaling represents a major cellular stress response, numerous investigations have focused on the role of the JNK pathway signaling in modulating the cardiac response to external stresses such as pressure overload. In this context, a key downstream target of JNK, transcription factor c-Jun, appears important for some cardiac hypertrophy responses. For example, c-Jun mediates insulin-like growth factor (IGF)-Akt signaling which leads to the development of pathologic hypertrophy and heart failure, whereas inhibition of c-Jun signaling inhibits cardiac hypertrophy [105]. The study by Choukroun et al. (1999) [103] was one of the first to report that expressing a dominant-negative upstream activator of JNK (SEK-1/MKK4) prevented both JNK activation and cardiac hypertrophy in response to pressure overload. Further, overexpression of a dnJNK inhibited cardiac hypertrophy a rat LV pressure overload model, through a FOXO3a-mediated mechanism [106]. Consistent with these results, transgenic mice with cardiac-specific overexpression of an activated form of another upstream JNK-activator, MKK7 (Ser^281^ and Thr^275^ to Asp, called MKK7D) exhibited a specific increase in cardiac tissue JNK1 and JNK2 activity, without activation of ERK or p38 [107]. These cardiac MKK7D mice died at approximately 7 weeks of age with concomitant evidence of congestive heart failure, suggesting that excess JNK1/2 activation promoted this adverse cardiac phenotype. In a recent study, JIP3 deficiency protected against cardiac hypertrophy with suppression of myocardial inflammation, oxidative stress, fibrosis accumulation, and ER stress in a mouse model of cardiac hypertrophy. The protective effects of JIP3 knockout on cardiac hypertrophy appeared linked to the inactivation of the JNK pathway [108]. Collectively, these findings suggest that chronic activation of JNK enzymatic activity promotes pathologic cardiac hypertrophy in comparison with inhibition of this signaling which opposes this process.

However, several studies suggest alternative effects of JNK on cardiac hypertrophy and on LV failure. Whole body JNK1^−/−^ mice exhibited an abnormal response to pressure overload by transverse aortic constriction manifested by reduced LV systolic function within 3 days after pressure overload, which lasted for several weeks before eventually normalizing. Further, JNK1 deletion did not affect the overall hypertrophic response, though LVs of JNK1^−/−^ mice did display early cellular apoptosis. These findings suggest that JNK1 plays a protective role to maintain LV systolic function in the acute phase after pressure overload [109]. Interestingly, JNK1DN mice, as well as combined JNK1^+/−^; JNK2^−/−^ mice developed increased early LV hypertrophy after only 3 days of pressure overload, with increased mortality observed in the JNK1^+/−^; JNK2^−/−^ mice in the chronic setting, further supporting a protective role of JNK signaling after pressure overload [110]. These two strains also developed baseline LV hypertrophy with aging, though in these experiments the investigators did not perform LV functional or hemodynamic studies, making it difficult to interpret whether the hypertrophy and mortality arose from intrinsic CM process, or rather from potential external stress such as chronic hypertension. Mechanistic studies did demonstrate, however, that disruption of JNK promoted increased myocardial NFAT activation as assessed by NFAT-luciferase assay, suggesting that JNK normally opposes CM pathologic NFAT signaling.

The effects of CM-specific deletion of MAP2Ks (MKK7 and MKK4) have more recently been investigated in models of heart failure. Transgenic mice with cardiac-specific overexpression of an MKK7-JNK1 fusion protein (which behaves like a constitutively active JNK) had normal ventricular weight at baseline but were resistant to stress-induced cardiac hypertrophy [111]. Further, MKK7 deletion from the CM promoted increased arrhythmia vulnerability in hypertrophied hearts [110]. Additionally, in response to surgical pressure overload, mice with CM-specific MKK7 deletion developed severe LV hypertrophy, contractile dysfunction, LV dilation, and fibrosis within 1 week of pressure overload, with corresponding increased myocardial NFAT activation [111]. Similarly, CM-specific deletion of the other JNK-selective MAP2K, MKK4 produced a similar phenotype, with increased pressure overload-induced LV hypertrophy by 1 week post-TAC, which progressed to overt systolic and diastolic dysfunction after 5 weeks of TAC [112]. As with MKK7, CM deletion of MKK4 appeared to promote ventricular arrhythmia in mice, with altered expression and localization of the gap junction protein connexin 43 [113].

Similar discrepant results have been observed with studies of upstream JNK activators, including MAP3Ks. Whole body deletion of the MAP3K MEKK1 had no effect on the hypertrophic response to TAC, but led to worsening contractile dysfunction, LV dilation, and heart failure as evidenced by increased lung mass and mortality [114]. Another upstream JNK activator, CDC42, was identified in seminal studies as one of three myocardial-expressed genes modulated by the microRNA, miR-133 [115]. CM-specific deletion of CDC42 led to increased TAC-induced LV hypertrophy, reduced myocardial JNK activation, worsened systolic function, increased heart failure phenotype, and enhanced myocardial NFAT activation [116]. Importantly, crossing these mice with CM-specific MKK7 transgenic mice reduced the LV hypertrophic response, indicating that JNK deficiency likely mediated the consequences of CDC42 deletion from the CM (Figure 4).

CDC42 binds and induces autoactivation of another MAP3K, MLK3 [117]. MLK3 whole body knockout mice displayed baseline LVH with normal LV function [100]. In response to TAC, however, the MLK3^−/−^ mice developed more severe LV systolic and diastolic dysfunction, as well increased LV end diastolic pressures, indicating heart failure. By 1-month post-TAC the hearts of MLK3 knockout mice displayed more overt LV systolic dysfunction as well as a pattern of pathological gene expression. Chemical inhibition of MLK3 kinase activity also blunted JNK activation in cultured CMs, and induced myocyte hypertrophy, suggesting mechanistic effects of MLK3 specific to the CM.

Finally, though not typically considered a MAP3K, the cGMP-dependent protein kinase I α isoform promotes myocardial JNK activation after LV pressure overload. Mice with selective mutation in the PKGIα leucine zipper protein interaction domain developed striking early mortality after LV pressure overload, with increased lung mass indicating heart failure [118]. Interestingly, activation of MKK4 and of JNK was blunted in LVs of these mice as early as 2 days post-pressure overload. This increased mortality suggests that these mice suffered from impaired PKG-mediated MKK4-JNK activation.

Several themes emerge from the above studies. First, though the precise roles of JNK signaling in the LV hypertrophic process remain unclear, and are likely highly complex, many of these studies identify a critical role of JNK pathway signaling in promoting and preserving the LV functional response to stress (particularly to pressure overload). The increased lung mass, LV end diastolic pressures, and mortality observed in some of these studies further support that disruption of JNK signaling promotes features of the heart failure phenotype. A number of these studies suggest a key role of cardiomyocyte JNK signaling in preserving LV functional response early after pressure overload. Second, enhanced myocardial cell apoptosis and increased NFAT activation appear prominent in these models, suggesting that JNK opposes the pressure overload-induced cellular apoptosis and pathological NFAT activation.

Several possibilities may underlie these apparently conflicting findings on the role of JNK signaling in the hypertrophic and cardiac functional response to stress. One explanation could be that different upstream stress signals, by activation of different MAP3Ks, target various cellular compartments of JNK, thus lead to regulation of different pools of JNK substrates. As a specific example, JNK phosphorylation of nuclear NFAT promotes pathological NFAT activation. However, JNK phosphorylation of NFAT outside of the nucleus prevents its nuclear import and thus inhibits NFAT-mediated gene expression. It is possible to envision a balance of these opposing mechanisms which could be altered depending on the specific cellular localization of JNK activation. In line with this, as described in detail in the introduction, different MAP3Ks and upstream JNK activators can be selectively modulated by separate extracellular signals (such as ROS and FFAs, for example). Further, though different MAP3Ks and MAP2Ks activate JNK, they do so in the context of specific scaffolding molecules, which likely have molecular and spatial specificity. In other words, MAP3K-MAP2K-JNK activation at one specific scaffold may target separate substrates and promote different responses than the JNK activation arising from a different scaffolding complex. Further experimental work will be required to test this hypothesis.

**Figure 5:**
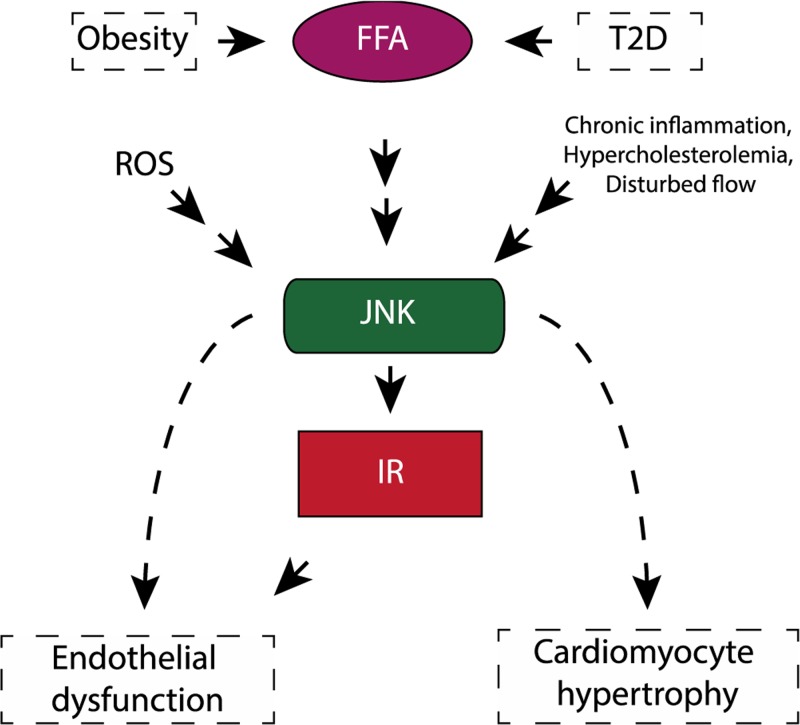
JNK in cardiometabolic diseases A schematic diagram explains the JNK connects the metabolic diseases to cardiovascular disease.

Finally, both the pro- and anti-hypertrophic evidence outlined above must also be interpreted within the limitations of existing genetic models. For example, *in vivo*, the JNK pathway provides a highly efficient rapid signaling system to transmit a broad variety of extracellular stresses into intracellular responses. Therefore, it remains unclear whether the findings from permanent genetic alteration models allow accurate interpretation of the likely finely tuned and temporally diverse actions of these kinases in the normal state. Moreover, as described above, permanent genetic deletion models, even if cell-specific, may induce splice variants of JNK, or could induce compensatory up-regulation of redundant enzymes, further confounding the phenotypic interpretation of these models. Finally, while the majority of the studies of upstream JNK activators provide correlative evidence of reduced JNK activity, they generally do not provide evidence that rescue of JNK can reverse the pathologic findings. Thus, the degree to which reduction in JNK actually mediates the above phenotypes remains unclear. For these reasons, further studies will be required to address these contradictory findings in new model systems. Using a conditional and temporal knockout of all three JNK isoforms in the heart could further elucidate JNKs role in cardiac hypertrophy and in the heart failure process. Finally, both inflammation and metabolic dysfunction within the myocardium have become increasingly recognized as contributing to cardiac hypertrophy and heart failure. However, the roles of JNKs in the regulation of cardiac metabolism, modulation of cardiac inflammation and immune cell honing to the myocardium remains largely unexplored. We therefore suggest that better understanding of JNK effects on the cardiac metabolism and inflammation will help clarify the complex role of this signaling axis in cardiac hypertrophy and failure (Figures 4 and 5).

## JNK in ischemic injury

Heart attack or acute myocardial infarction (AMI) affects more than 3 million Americans each year. AMI occurs when the blood flow to the heart muscle is cut off usually due to a blockage of the coronary arteries from atherosclerosis resulting in tissue ischemia. While restoration of blood flow (reperfusion) is necessary for survival, reperfusion itself causes significant injury and stimulates ROS production and inflammatory signaling. Under these conditions JNK is generally thought to promote injury in mouse [119]. However, it is also recognized that JNK can be protective if the period of ischemia is brief [46]. Utilizing a pharmacologic inhibitor, SP600125, JNK activity was necessary for recovery after brief ischemia reperfusion, but at longer time points conferred a destructive presence. This finding has demonstrated to specifically require JNK1 as JNK1^−/−^ mice demonstrated greater damage after brief ischemia. Therefore, JNK could be important for the phenomenon known as ‘ischemic preconditioning’ which describes a brief period of non-lethal injury or ischemia that can often render the tissue resistant to further injury. In the setting of myocardial infarction, inflammatory mechanisms are triggered by ischemic or necrotic myocardium, which leads to ventricular remodeling. If the remodeling is pathologic (involving ventricular dilation and thinning), it can worsen systolic function and, eventually, cause heart failure. In this context, ASK1-JNK1/2 signaling is thought to promote pathological cardiac remodeling after myocardial infarction. Mice lacking ASK1 exhibit reduced activation of JNK1/2, but not p38, in the infarct border zone [47]. This study suggests that ASK1 acts as a specific activator of JNK1/2 signaling in this particular context [70]. The ASK1-deficient mice had reduced cardiac remodeling, with reduced fibrosis in the border zone and remote myocardium, reduced diastolic LV dimension, improved fractional shortening, and reduced cardiomyocyte apoptosis in the border zone [48,49].

JNK activation has been reported in rat heart during exercise [120]. A recent study of a reperfusion injury model in rat has shown that scaffold JIP inhibitor, SU3327, does not have the ability to protect the heart from IR injury. Use of JIP inhibitor SU3327 during IR actually aggravated the cardiac dysfunction via mitochondrial dysfunction [121]

Another condition impacted by ischemia is peripheral artery disease (PAD). PAD affects more than 200 million people worldwide [122,123]. PAD occurs due to atherosclerotic occlusions of peripheral arteries, primarily of the legs and to a lesser extent, the arms [122,123] due to underlying risk factors (as described above). Therefore, JNK signaling is likely important for multiple steps of disease onset and progression. In the setting of PAD, JNK1 and JNK2 deficiency specifically in the endothelium resulted in impaired blood flow recovery [50] after induction of a mouse model of PAD (hind limb ischemia; HLI). The present study found JNK is required for collateral vessels formation during development which is essential in the initial recovery of HLI. The present study showed that the Notch-JNK pathway was a prerequisite for proper development of collaterals in the hindlimb and concludes that JNK plays a critical role in vascular development, which can affect vascular disease outcomes such as PAD. However, the role of JNK in PAD requires further investigation as the role of JNK in macrophages or other cell types important for mediating blood flow recovery has not yet been investigated.

## Cardiovascular JNK biology in CMS: suggestions for research directions

In humans, CMS characteristics of chronic inflammation, insulin resistance, and metabolic abnormalities contribute to atherosclerosis and cardiac hypertrophy. These in turn lead to consequences such as heart failure and myocardial infarction. The studies discussed in this review establish the JNK signaling pathway as an essential regulator of both CMS and of its consequent conditions. However, while studies in genetically modified mice nearly uniformly support that JNK signaling promotes CMS itself, the findings in studies of CMS consequences such as atherosclerosis and heart failure have been less uniform.

We suggest several limitations in the current knowledge which if addressed could provide a more holistic understanding of the functions of JNK in CMS-related diseases. First, future studies using mutants of upstream MAP3Ks and MAP2Ks, will be beneficial from investigating a requirement of JNK for the different phenotype in mouse and other animal models. For example, while MLK3 deletion correlates with reduced JNK activation as well as a number of phenotypes of JNK deficiency [100], in many cases the ability of JNK activation to rescue the phenotypes remains unknown. In addition, MLKs and other JNK activating proteins modulate substrates other than JNK through both kinase dependent and independent mechanisms. Therefore, clarifying JNK-dependent versus JNK-independent cardiovascular actions of these molecules should be a scientific goal.

Second, the conflicting findings outlined above must be interpreted within the limitations of existing genetic models. For example, *in vivo*, the JNK pathway provides a highly efficient rapid signaling system to transmit a broad variety of extracellular stresses into intracellular responses. Therefore, it remains unclear whether the findings from permanent genetic alteration models, even if tissue-specific, allow accurate interpretation of the likely fine-tuned and temporally diverse actions of these kinases in the normal state. Moreover, genetic deletion models, even if cell-specific, may induce splice variants of JNK, or could induce compensatory up-regulation of redundant enzymes, further confounding the phenotypic interpretation of these models. Ideally, future studies can measure JNK activation across multiple time-points and cell types throughout given disease processes such as atherosclerosis or heart failure.

From the potential therapeutic perspective, several questions deserve further investigation. First, does modulation of JNK have more potential benefit for acute events (heart attack, decompensated heart failure), or for chronic conditions such as obesity, insulin resistance, and chronic inflammation? As a related question, can modulation of JNK in CMS actually prevent downstream acute disease events? Finally, for CMS phenotypes, is it more efficacious to target upstream JNK activators like MLKs or MAP2Ks, instead of direct modulation of JNK? These questions remain unanswered but could be straightforward to test.

## Concluding remarks

This review summarizes the role of the JNK family in cardiovascular pathology. JNK signaling has emerged as main regulator of cytokine production and inflammation during obesity and chronic metabolic stress condition. Modulation of the prolonged activation of the JNK pathway will be beneficial during deferent metabolic diseases like diabetes. Diabetic patients are four times more likely to develop cardiovascular diseases than its controls. Therefore, it would be natural to predict that controlling the JNK pathway will be beneficial to this group of patients.

Though previously published data are inconsistent on the role of JNK in animal models of atherosclerosis, we cannot rule out that JNK plays a different role in different cell types. Therefore, there is a possibility that the phenotype of JNK deletion in the immune cells differ from the endothelial cells. Future studies would be optimal to clarify this inconsistency by using conditional or inducible knockout animal models.

As data suggest, JNK plays a key role in obesity-induced pro-inflammatory macrophage polarization and insulin resistance development. JNK activity also seems to influence cardiac remodeling after ischemic injury/MI. These various observations are likely the result of discrete mechanisms stemming from stimuli-specific activation of the fifteen different upstream MAP3K activators of JNK together with several possible combinations of various scaffold proteins in the signaling complex (Figure 1). As JNK1 and JNK2 have overlapping functions, using tissue-specific mouse models are needed for a comprehensive understanding of the JNK family members’ role both in cardiovascular diseases and to determine whether there is therapeutic potential in blocking these pathways in models of cardiovascular pathology (Figure 5).

Recently the role of JNK has been implicated in angiogenesis during development. Nonetheless it appears that JNK plays a minor role in adult angiogenesis, though it is known that JNK is required for apoptosis during prolonged stress. Although this is not clear, one can predict that JNK can be required for tumor elimination.

## Abbreviations

AAA: abdominal aortic aneurysm
AgRP: Agouti-related peptide
AMI: acute myocardial infarction
ApoE^−/−^: Apo lipoprotein E knockout mouse
AP1: Activator protein 1
ASK: apoptosis signal-regulating kinase
ATF2: Activating transcription factor 2
A-FABP: adipocyte fatty acid binding protein
CDC42: Cell division protein 42
CM: cardiac myocyte
CMS: cardiometabolic syndrome
CNS: Central nervous system
COPD: chronic obstructive pulmonary disease
dnJNK: dominant-negative JNK
FFA: free fatty acid
HFD: high-fat diet
HLI: hind limb ischemia
IL6: Interleukin 6
JIP: JNK-interacting protein
JNK: c-Jun N-terminal Kinase
LDL: low-density lipoprotein
LDLR^−/−^: LDL receptor knockout
MEKK: Mitogen-Activated Protein Kinase Kinase Kinase
MKP-1: MAP kinase phosphatase-1
MLK: mixed lineage kinase
NFAT: Nuclear factor of activated T-cells
PAD: peripheral artery disease
ROS: reactive oxygen species
TAK1: transforming growth factor β-activated kinase 1
TNF: Tumor necrosis factor
